# Supplementary figures and images for: Future sea-level rise drives rocky intertidal habitat loss and benthic community change
Source: PeerJ. 2020 May 29;8:e9186. doi: 10.7717/peerj.9186 (PMC7263295; doi:10.7717/peerj.9186)

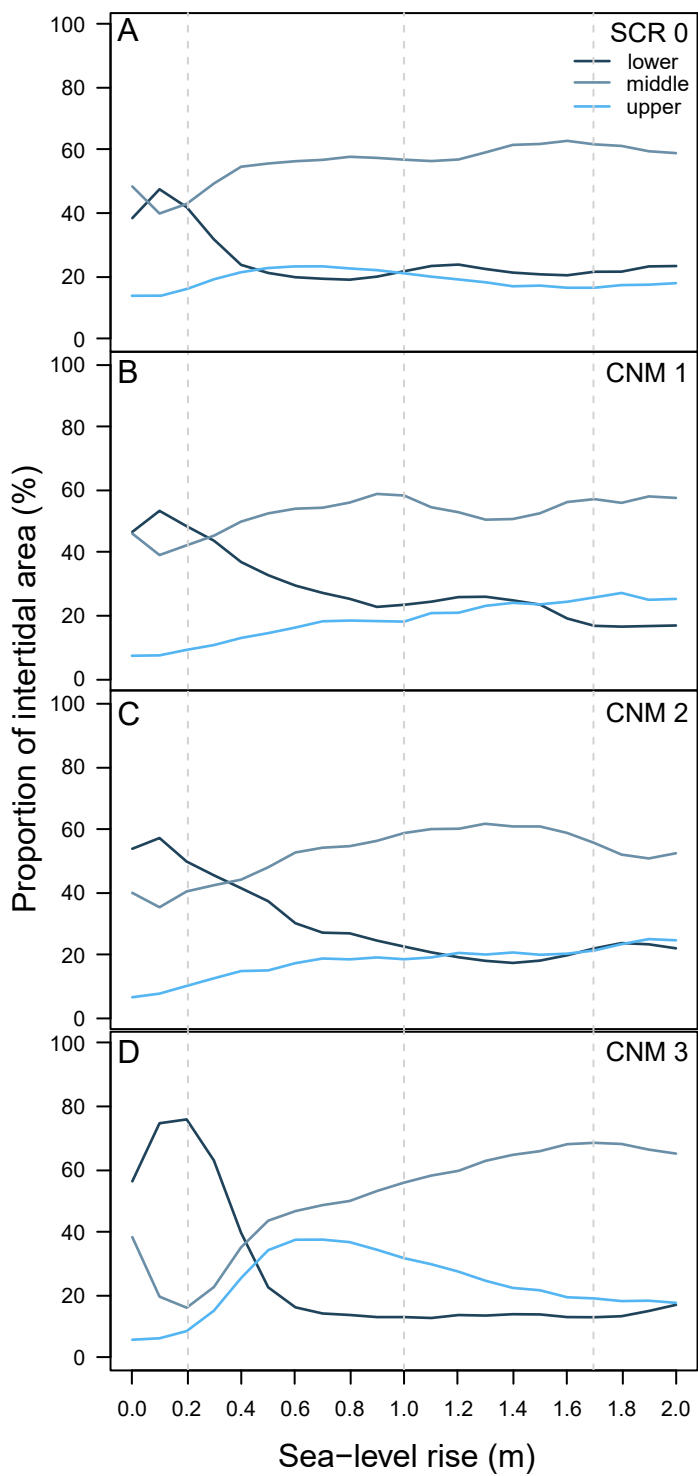

Supplement: Figure S1 — Proportion of intertidal area contributed by each tidal zone (lower, middle, and upper) under 0–2.0 m of sea-level rise for survey sites (A) Scripps Coastal Reserve (SCR 0), (B) Cabrillo National Monument 1 (CNM 1), (C) Cabrillo National Monument 2 (CNM 2), and (D) Cabrillo National Monument 3 (CNM 3). [file peerj-08-9186-s004.pdf]
