# Supplementary material for: Future sea-level rise drives rocky intertidal habitat loss and benthic community change
Source: PeerJ. 2020 May 29;8:e9186. doi: 10.7717/peerj.9186 (PMC7263295; doi:10.7717/peerj.9186)
Supplement: Table S1 — Tidal datums were used to segment the intertidal into zones (upper, middle, lower). Data presented in m in reference to MLLW. Upper Zone Boundaries: HAT–MHW Middle Zone Boundaries: MHW–MLW Lower Zone Boundaries: MLW–MLLW. [file peerj-08-9186-s005.docx]

**S1 Table**

| Station ID | 9410230 | 9410170 | Tidal Datum: | Tidal Zone Designation: | Representative Target Species: |
| --- | --- | --- | --- | --- | --- |
| Tidal Datum: | La Jolla | San Diego | Tidal Epoch: 1983 -2001, Units: m above MLLW |  |  |
| HAT | 2.177 | 2.354 | Highest Astronomical Tide | Upper / High Intertidal | Goose Barnacles |
| MHHW | 1.624 | 1.745 | Mean Higher High Water |  | Owl Limpets |
| MHW | 1.402 | 1.52 | Mean High Water |  | Barnacles |
| DHQ | 0.222 | 0.225 | Mean Diurnal High Water Inequality | Middle Intertidal | Mussels |
| MSL | 0.832 | 0.897 | Mean Sea Level (mean of hourly heights) |  | Rockweeds |
| DLQ | 0.276 | 0.285 | Mean Diurnal Low Water Inequality |  | Red Algal Turf |
| MLW | 0.276 | 0.286 | Mean Low Water | Lower / Low Intertidal | Surfgrass |
| MLLW | 0 | 0 | Mean Lower Low Water |  | Brown Macroalgae (Kelps) |
| LAT | -0.573 | -0.644 | Lowest Astronomical Tide |  |  |

Upper Zone Boundaries: HAT – MHW

Middle Zone Boundaries: MHW – MLW

Lower Zone Boundaries: MLW - MLLW
