# Supplementary material for: Future sea-level rise drives rocky intertidal habitat loss and benthic community change
Source: PeerJ. 2020 May 29;8:e9186. doi: 10.7717/peerj.9186 (PMC7263295; doi:10.7717/peerj.9186)
Supplement: Table S2 — Table shows species and functional group level identifications used for stratified random point counts and invertebrate counts. [file peerj-08-9186-s006.docx]

**S2 Table**

| code | common name | genus | species | functional group |
| --- | --- | --- | --- | --- |
| r | Rock | *NA* | *NA* | abiotic factor |
| s | Sand | *NA* | *NA* | abiotic factor |
| ac | Articulated Coralline | *Corallina / Jania* | *spp.* | algae - calcified |
| cc | Crustose Coralline Algae | *NA* | *NA* | algae - calcified |
| ta | Non-Coralline Crust | *NA* | *NA* | algae - encrusting |
| cp | *Colpomenia* | *Colpomenia* | *peregrina* | algae - macroalgae |
| dd | *Dictyota* | *Dictyota* | *spp.* | algae - macroalgae |
| du | *Dictyopteris* | *Dictyopteris* | *undulata* | algae - macroalgae |
| eb | *Petalonia* | *Petalonia* | *binghamiae* | algae - macroalgae |
| em | Feather Boa Kelp | *Egregia* | *menziesi* | algae - macroalgae |
| en | Seamoss | *Caulacanthus* | *ustulatus* | algae - macroalgae |
| gr | *Gelidium robustum* | *Gelidium* | *robustum* | algae - macroalgae |
| po | Nori | *Pyropia* | *perforata* | algae - macroalgae |
| pp | *Plocamium* | *Plocamium* | *pacificum* | algae - macroalgae |
| ra | Red Algae | *unk.* | *unk.* | algae - macroalgae |
| sc | Golden Rockweed | *Silvetia* | *compressa* | algae - macroalgae |
| sm | Wireweed | *Sargassum* | *muticum* | algae - macroalgae |
| ss | Chainbladder Kelp | *Stephanocystis* | *spp.* | algae - macroalgae |
| ul | Sea Lettuce | *Ulva* | *spp.* | algae - macroalgae |
| wr | Wrack | *NA* | *NA* | algae - macroalgae |
| zf | Zonaria | *Zonaria* | *farlowii* | algae - macroalgae |
| gt | Green Algal Turf | *NA* | *NA* | algae - turf |
| tu | Turf | *NA* | *NA* | algae - turf |
| ch | Chiton | *unk.* | *unk.* | invertebrate - motile |
| lg | Owl Limpet | *Lottia* | *gigantea* | invertebrate - motile |
| li | Periwinkle | *Littorina* | *spp.* | invertebrate - motile |
| lo | Limpet | *Lottia* | *spp.* | invertebrate - motile |
| nu | Dog Whelk | *Nucella* | *spp.* | invertebrate - motile |
| tf | Turban Snail | *Tegula* | *funebralis* | invertebrate - motile |
| NA | NA | *NA* | *NA* | NA |
| sg | Surfgrass | *Phyllospadix* | *scouleri / torreyi* | plant |
| ae | Clonal Anemone | *Anthopleura* | *elegantissima* | invertebrate - sessile |
| as | Green Anemone | *Anthopleura* | *sola* | invertebrate - sessile |
| ba | Barnacle | *Chthamalus / Balanus* | *NA* | invertebrate - sessile |
| gb | Goose Barnacle | *Pollicipes* | *polymerus* | invertebrate - sessile |
| lb | Large Barnacle | *NA* | *NA* | invertebrate - sessile |
| mc | California Mussel | *Mytilus* | *californianus* | invertebrate - sessile |
| pc | Honeycomb Tube Worm | *Phragmatopoma* | *californica* | invertebrate - sessile |
| tr | Pink Barnacle | *Tetraclita* | *rubescens* | invertebrate - sessile |
